# Targeting GABA_A_R-Associated Proteins: New Modulators, Labels and Concepts

**DOI:** 10.3389/fnmol.2019.00162

**Published:** 2019-06-26

**Authors:** Vladimir Khayenko, Hans Michael Maric

**Affiliations:** ^1^Institute of Structural Biology, Rudolf Virchow Center for Experimental Biomedicine, University of Würzburg, Würzburg, Germany; ^2^Department of Biotechnology and Biophysics, Biocenter, University of Würzburg, Würzburg, Germany

**Keywords:** GABA_A_ receptors, gephyrin, collybistin, protein-protein interaction (PPI), super resolution microscopy, fluorescent probes, dimeric peptide, peptide inhibitor design

## Abstract

γ-aminobutyric acid type A receptors (GABA_A_Rs) are the major mediators of synaptic inhibition in the brain. Aberrant GABA_A_R activity or regulation is observed in various neurodevelopmental disorders, neurodegenerative diseases and mental illnesses, including epilepsy, Alzheimer’s and schizophrenia. Benzodiazepines, anesthetics and other pharmaceutics targeting these receptors find broad clinical use, but their inherent lack of receptor subtype specificity causes unavoidable side effects, raising a need for new or adjuvant medications. In this review article, we introduce a new strategy to modulate GABAeric signaling: targeting the intracellular protein interactors of GABA_A_Rs. Of special interest are scaffolding, anchoring and supporting proteins that display high GABA_A_R subtype specificity. Recent efforts to target gephyrin, the major intracellular integrator of GABAergic signaling, confirm that GABA_A_R-associated proteins can be successfully targeted through diverse molecules, including recombinant proteins, intrabodies, peptide-based probes and small molecules. Small-molecule artemisinins and peptides derived from endogenous interactors, that specifically target the universal receptor binding site of gephyrin, acutely affect synaptic GABA_A_R numbers and clustering, modifying neuronal transmission. Interference with GABA_A_R trafficking provides another way to modulate inhibitory signaling. Peptides blocking the binding site of GABA_A_R to AP2 increase the surface concentration of GABA_A_R clusters and enhance GABAergic signaling. Engineering of gephyrin binding peptides delivered superior means to interrogate neuronal structure and function. Fluorescent peptides, designed from gephyrin binders, enable live neuronal staining and visualization of gephyrin in the post synaptic sites with submicron resolution. We anticipate that in the future, novel fluorescent probes, with improved size and binding efficiency, may find wide application in super resolution microscopy studies, enlightening the nanoscale architecture of the inhibitory synapse. Broader studies on GABA_A_R accessory proteins and the identification of the exact molecular binding interfaces and affinities will advance the development of novel GABA_A_R modulators and following *in vivo* studies will reveal their clinical potential as adjuvant or stand-alone drugs.

## Introduction

γ-aminobutyric acid type A receptors (GABA_A_Rs) are the principal mediators of phasic and tonic inhibition in the human brain, being a vital part of the molecular machinery that creates cognition, behavior, and consciousness (Fritschy and Panzanelli, 2014). Dysfunctional GABA_A_Rs are involved in the pathogenesis of neurodevelopmental disorders (Ali Rodriguez et al., 2018), schizophrenia (de Jonge et al., 2017), postpartum depression (Mody, 2019), epilepsy (Palma et al., 2017; Hines et al., 2018), Alzheimer’s disease (Govindpani et al., 2017), autism (Vien et al., 2015) and stroke (Darmani et al., 2016; Wang et al., 2018). Structurally, these receptors belong to the pentameric ligand-gated ion channels harboring an extracellular domain (ECD), followed by four helical transmembrane domains (TMDs) and loops connecting these helices. GABA_A_Rs display a highly subtype-specific cellular and sub-cellular distribution and exhibit distinct physiological properties, making them very attractive pharmaceutical targets.

**Figure 1:**
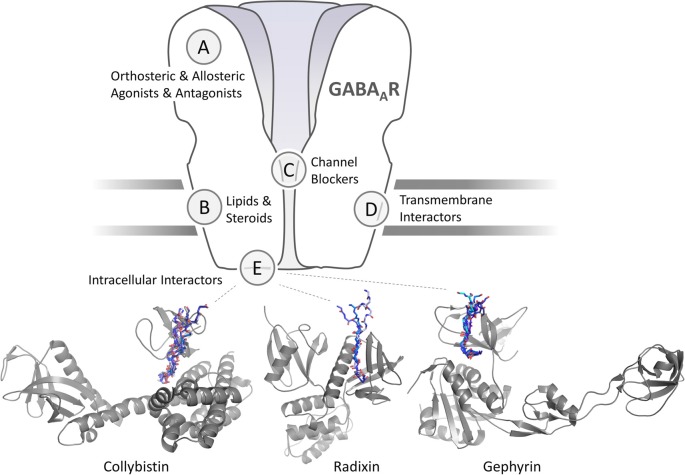
Schematic representation of a γ-aminobutyric acid type A receptors (GABA_A_Rs) and sites for pharmaceutical intervention. Orthosteric and allosteric agonists and antagonist are classical activity modulators that bind directly to the extracellular domain (ECD) or, in case of lipids and neurosteroids to the transmembrane domain (TMD) of GABA_A_Rs. Each of the sites could occur in five subunits or at five interfaces, or only in distinct subunits and specific interfaces. Channel blockers bind within the ion pore formed by the GABA_A_R pentamer. Intracellular interactors such as Collybistin (CB; Hines et al., 2018) and gephyrin (Maric et al., 2014) interact with distinct intracellular regions of a subset of GABA_A_R subunits. Transmembrane interactors such as LHFPL4 interact with the TMDs of γ2 subunit containing GABA_A_Rs (Davenport et al., 2017; Yamasaki et al., 2017). Cartoon representation of structurally characterized and predicted scaffold-GABA_A_R complexes. CB is shown in gray in its extended conformation (PDB-ID 4mt7) with its SH3 domain (PDB-ID 4mt6; Soykan et al., 2014) binding to a fragment of the GABA_A_R α2 subunit (Hines et al., 2018). Peptide backbones of resolved SH3 domain ligands (PDB-IDs 2df6, 4hvu, 4hvv, 4hvw, 4j9f, 4ln2 and 4rtz) are superimposed to indicate the putative GABA_A_R α2 binding site. The radixin FERM domain is shown in gray. Peptide backbones of resolved radixin FERM domain ligands (PDB-IDs 1j19, 2ems, 2d2q) are superimposed to indicate the putative GABA_A_R α5 binding site. Cartoon representation of the gephyrin E domain in complex with short linear GABA_A_R (PDB-IDs 4tk1, 4tk2, 4tk3, 4tk4) and GlyR derived peptides (PDB-IDs 2fts, 4u90, 4u91).

First GABA_A_R targeting compounds have been discovered more than a century ago. In 1904, Bayer marketed barbital, the first barbiturate and positive allosteric modulator of GABA_A_Rs (Löscher and Rogawski, 2012). In the 1960s, benzodiazepines, a new class of GABA_A_R allosteric modulators (Sancar and Czajkowski, 2011), became commercially available. Today, modulators of GABA_A_R activity find broad clinical use as anesthetics (Propofol; Olsen, 2018), anticonvulsants (Gabapentin) or as hypnotics, muscle-relaxants and anxiolytics (Clonazepam, Diazepam), and new experimental medicines are developed. Nonetheless, wider application of these classical GABA_A_R modulators is limited by their lack of receptor subtype specificity, due to the fundamental structural and functional constraints: pharmacologically exploited sites are small hydrophobic pockets with high subunit sequence homology located at the folded ECDs and TMDs of the ion channels (Figure 1; Miller et al., 2017; Kasaragod and Schindelin, 2018; Masiulis et al., 2019). Additionally, binding sites on the interface between two subunits, such as the benzodiazepine binding site, are shared among different synaptic receptor subtypes. Consequently, the action of classical clinically relevant GABA_A_R ligands can be unspecific and provoke unavoidable side effects.

Molecules modulating receptor signaling through accessory proteins in the central nervous system (CNS; Figure 1) emerged as a new class of pharmaceuticals with superior receptor specificity and potential to treat epilepsy, neuropathic pain, fibromyalgia, migraines, and other diseases (Maher et al., 2017). Therefore, targeting GABA_A_R-associated proteins might be a superior pharmacological strategy compared to the classical approaches. This rational approach, however, requires detailed knowledge and advanced understanding of the intracellular signaling of distinct GABA_A_R subtypes. The large number of post-synaptic candidate proteins that directly or indirectly associate with GABA_A_Rs is still increasing (Krueger-Burg et al., 2017), with functional studies exploring some of their physiological roles and organization (Uezu et al., 2016; Lu et al., 2017), yet, the specific molecular details of these interactions remain largely unknown. We hypothesize that the identification of the exact molecular binding interfaces and binding affinities of known and newly identified GABA_A_R associated proteins will not only greatly expand our basic understanding of CNS function, but also provide new pharmaceutical opportunities.

## Adjusting GABAergic Signaling Through Intracellular Modulation

The majority of GABA_A_Rs assemble as heteropentamers to form GABA-gated chloride channels. Different subunit combinations possess unique pharmacology (Olsen and Sieghart, 2009), divergent brain region distribution (Wisden and Seeburg, 1992), cell-type specific expression (Lee and Maguire, 2014), and varying subcellular localization between synaptic and extra-synaptic sites (Mody and Pearce, 2004). Thus, subtype-specific modulators of GABA_A_R signaling should affect distinct circuits, brain regions or subcellular populations with improved accuracy and more selective pharmacology. Combined structural and functional studies have revealed the molecular details of the interplay of the ECD and TMDs in channel gating (Miller and Aricescu, 2014; Lu et al., 2017; Kasaragod and Schindelin, 2018; Zhu et al., 2018; Laverty et al., 2019). Structural studies of the receptors could, so far, not resolve most of the presumably intrinsically disordered intracellular regions of GABA_A_Rs. Short intracellular receptor regions, however, do adopt defined conformations when engaged with structured intracellular interactors, such as gephyrin (Maric et al., 2014) and the AP2 complex (Kittler et al., 2008; Table 1). Functional studies validated that distinct motifs within these unstructured regions exert tight control over channel biosynthesis, recycling, diffusion and synaptic recruitment (Tretter et al., 2012; Nakamura et al., 2015; Groeneweg et al., 2018; Lorenz-Guertin and Jacob, 2018). Remarkably, these intracellular regions display the highest level of sequence heterogeneity among receptor subunits, thereby enabling subtype-specific modulation of GABAergic signaling. Agents targeting these discrete regions will probably be highly selective and could affect GABA_A_R subtypes with distinct functional and pharmacological properties. It is noteworthy that, so far, all intracellular GABA_A_R interactions that displayed sufficient affinity and specificity ended up being exploited to modulate neuronal communication (Table 1).

**Table 1:** List of all known intracellular GABA_A_R-associated proteins that display micromolar or better binding affinity.

GABA_A_R Interactor	GABA_A_R Subunit	Mapped Binding Site	Affinity [μM]	PDB ID	Physiological function	Inhibition or Interference
Gephyrin	α1	LIKKNNTYAPTATSYT^1^	17^7^	-	Clustering of distinct subsets of GABA_A_Rs at post-synaptic sites.^1,2,3,7^	Redistribution of post-synaptic GABAARs towards extra-synaptic sites. Decreased amplitude and frequency of phasic inhibitory currents.
	α3	FNIVGTTYPIN^2^	5^3,7^	4TK1^8^		
				4U90^9^		
	β2	AGLPRHSFGRNALERHVAQKKSRL^3^	17^3^	-		
AP-2	β3	KTHLRRRSS^4^	1^4^	-	Surface stabilization; increased receptor numbers, enhanced inhibitory post-synaptic currents^5^	Surface stabilization; increased receptor numbers, enhanced inhibitory post-synaptic currents^5^
	γ2	YECL^5^	0.4^5^	2PR9^5^		
Collybistin	α2	VMIQNNAYAVAVANYAPNL^6^	1^6^	-	Clustering of α2 subunit containing GABA_A_Rs at post-synaptic sites. Pronounced importance for receptors at the axon initial segment.^6^	Reduced GABA_A_R α2 cluster size and loss of GABA_A_R α2 subunit containing receptors. Reduced inhibitory post synaptic current amplitudes and decay times. Anxiety and seizure susceptibility.^6^

*Interacting GABA_A_R subunits, mapped binding sites, determined affinities and PDB-IDs of structures are listed as well as the reported function and the consequences of an acute inhibition or genetic interference. ^1^(Mukherjee et al., 2011); ^2^(Tretter et al., 2011); ^3^(Kowalczyk et al., 2013); ^4^(Kittler et al., 2005); ^5^(Kittler et al., 2008); ^6^(Hines et al., 2018); ^7^(Maric et al., 2011); ^8^(Maric et al., 2014); ^9^(Maric et al., 2015)*.

## Affecting Postsynaptic GABA_A_R Accumulation by Targeting Intracellular Scaffolds

**Figure 2:**
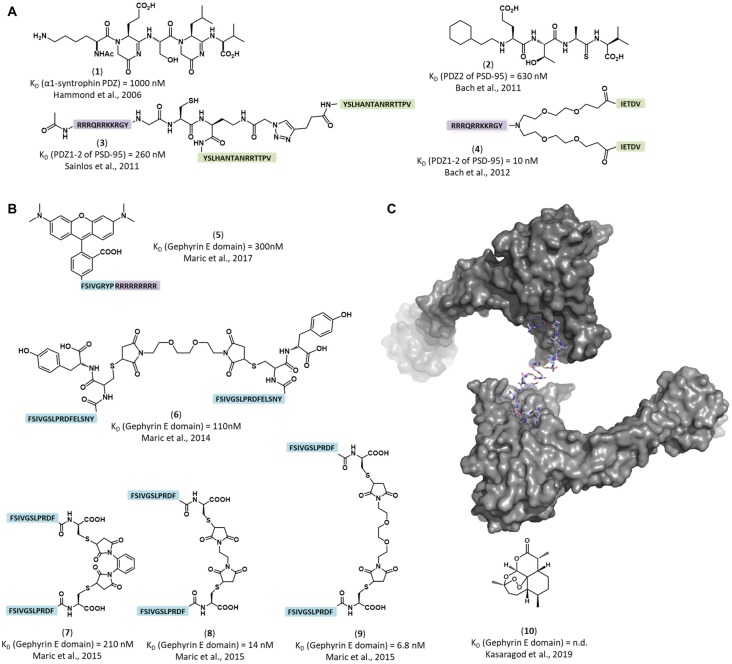
Structure of representative inhibitors and labels of the inhibitory post-synaptic scaffold gephyrin and the excitatory post-synaptic scaffold PSD-95. **(A)** Peptidomimetic and dimeric PSD-95/Discs-large/ZO-1 (PDZ) domain binders. PSD-95 binding peptides (green) were dimerized and combined with cell-penetrating moieties (violet). **(1)** Peptidomimetic ligand for a α-1 syntrophin PDZ domain (Hammond et al., 2006). **(2)** Peptidomimetic inhibitor of the PSD-95 PDZ2 domain (Bach et al., 2011) **(3)** Dimeric inhibitor of PSD-95 PDZ domains, showed strong inhibition of AMPA receptor synaptic currents (Sainlos et al., 2011). **(4)** Dimeric inhibitor of PSD-95 PDZ domains, showed neuroprotective properties in mice with cerebral ischemia (Bach et al., 2012). **(B)** Engineered peptide-based gephyrin inhibitors and fluorescent labels. **(5)** Peptide-based fluorescent gephyrin label (Maric et al., 2017). Tamra dye was directly conjugated to gephyrin binding sequence (blue) combined with cell penetrating peptide (in purple). **(6–9)** Nanomolar affinity dimerized gephyrin peptide binders (blue; Maric et al., 2015). **(10)** The small molecule inhibitor artemisinin competes with the universal engineered peptide-based molecules for receptor binding pocket (Kasaragod et al., 2019). **(C)** Representative crystal structure of a peptide dimer engaging with a gephyrin E domain dimer (PDB-ID 4U90; Maric et al., 2015).

The concept of neurotransmission modulation through targeting receptor-scaffolding protein interactions originated from studies investigating PSD-95/Discs-large/ZO-1 (PDZ) domain carrying proteins. These showed that through modulation of receptor-scaffolding protein interactions a variety of responses could be achieved, ranging from disruption of glutamate signaling to neuroprotective effects in ischemic brain damage (Hammond et al., 2006; Sainlos et al., 2011; Bach et al., 2012; Figure 2A). These results suggested that modulation of the inhibitory neurotransmission could be accomplished in a similar way, a concept recently proved with the inhibitory scaffold protein gephyrin (Maric et al., 2017).

Scaffolding proteins, such as gephyrin (Specht et al., 2013), radixin (Loebrich et al., 2006; Hausrat et al., 2015) and (collybistin, CB; Mayer et al., 2013; Hines et al., 2018), dynamically regulate the cell membrane distribution of postsynaptic and extrasynaptic GABA_A_Rs. Interestingly, their scaffolding functions are highly receptor specific, potentially allowing a fine tuning of neurotransmission.

### Radixin

Radixin is involved in the anchoring of numerous membrane proteins to the actin cytoskeleton (Kawaguchi et al., 2017). Its C-terminal domain mediates actin binding, while the N-terminal FERM domain functions as a universal protein-binding module that directly interacts with receptors, extracellular matrix components, transmembrane and adhesion proteins (Kitano et al., 2006; Takai et al., 2007; Terawaki et al., 2007, 2008; Yogesha et al., 2011; Figure 1). Radixin also harbors a central α-helical domain, which either adopts a closed or elongated conformation to allow its auto-inhibitory module to mask the FERM domain. In neurons, radixin is activated through phosphorylation, which enables its simultaneous binding to cytoskeletal elements and transmembrane proteins, including α5 subunit containing GABA_A_Rs (Loebrich et al., 2006; Hausrat et al., 2015). In primary hippocampal neuronal cultures, the association of radixin with α5-containing GABA_A_Rs at extrasynaptic sites decreases upon maturation, in contrast, the number of α5-containing GABA_A_Rs, that associate with gephyrin at post-synaptic sites remains constant (Brady and Jacob, 2015). Structural and thermodynamic details of the radixin-GABA_A_R α5 complex will reveal whether modulation can be achieved without simultaneously affecting the binding of other ligands.

### Collybistin

Collybistin (CB) is a guanine nucleotide exchange factor for Cdc42, a gephyrin binding partner (Kins et al., 2000) and an important determinant of inhibitory postsynaptic membrane formation and plasticity (Harvey et al., 2004; Tyagarajan et al., 2011a). Robust evidence supports the role of CB in GABA_A_R scaffolding with yeast three-hybrid studies (Saiepour et al., 2010) and proteomic studies (Nakamura et al., 2016) suggesting the presence of a tripartite complex between gephyrin, CB and α2 subunit containing GABA_A_Rs. Recently, a thermodynamic analysis revealed that CB binds GABA_A_R α2-subunits with high selectivity and affinity (Hines et al., 2018; Figure 1 and Table 1). CB is targeted to the neuronal surface membrane through phosphoinositides (Reddy-Alla et al., 2010; Ludolphs et al., 2016) and interfering human mutations result in cognitive deficits (Long et al., 2016; Chiou et al., 2019). Deficiency in CB reduces gephyrin and GABA_A_R clustering and impairs spatial learning (Papadopoulos et al., 2007, 2008). Moreover, mice with a mutation in the α2-subunit binding region of CB display a loss of a distinct subset of inhibitory synapses and a decreased amplitude of inhibitory synaptic currents, which results in a phenotype with increased susceptibility to seizures and early mortality (Hines et al., 2018). Notably, treatment with the α2/α3-selective positive modulator AZD7325 improves the conditions of affected mice, suggesting that compounds targeting the CB-GABA_A_R α2 complex could provide an alternative route to specifically affect GABA_A_Rs containing the α2 subunit.

### Neuroligin 2

Proteomic studies (Kang et al., 2014; Nakamura et al., 2016) revealed that the synapse-specific adhesion molecule neuroligin 2 (NL2; Varoqueaux et al., 2004) strongly associates with a subset of GABA_A_R subtypes and GABA_A_R scaffolds. Neuroligin dysfunction has been implicated in autism (Pettem et al., 2013) and specific intracellular residues in NL1 (Nguyen et al., 2016; Letellier et al., 2018) and NL2 (Poulopoulos et al., 2009; Kang et al., 2014) are critical for proper GABAergic signaling. Yet, the exact molecular interfaces, that mediate the direct or indirect gephyrin or CB dependent (Soykan et al., 2014) interactions of neuroligin with GABA_A_R, remain uncharacterized. These molecular insights could greatly contribute to our understanding of the development of the inhibitory synapse, as well as the underlying molecular causes of developmental diseases. Neuroligin family members exert distinct roles in the formation and stabilization of inhibitory and excitatory synapses and display distinct cellular and subcellular distributions. Accordingly, molecules that interfere with their isoform-specific interactions could act as highly cell-type selective modulators of neurotransmission.

### Gephyrin

Gephyrin is a prime candidate for the role of master regulator of neuronal function at inhibitory sites (Tyagarajan and Fritschy, 2014) and specifically the GABAergic synapses (Choii and Ko, 2015). Initially identified as a glycine receptor binding (Pfeiffer et al., 1982) and scaffolding protein (Feng et al., 1998), gephyrin was later found to be responsible for the post-synaptic accumulation of GABA_A_Rs. The loss of gephyrin clusters following the loss of the GABA_A_R γ2 subunit (Essrich et al., 1998) and the loss of GABA_A_R clusters upon gephyrin deficiency (Kneussel et al., 1999) substantiates their critical mutual dependency. Direct binding of gephyrin to α and β GABA_A_R subunits could be confirmed (Tretter et al., 2008, 2011; Maric et al., 2011; Mukherjee et al., 2011; Kowalczyk et al., 2013; Brady and Jacob, 2015), but the exact binding interfaces and affinities are still largely undefined. Structural, thermodynamic and high-end microscopic approaches elucidated the complex structure-function relationships between gephyrin and a distinct subgroup of inhibitory neurotransmitter receptors (Kasaragod and Schindelin, 2018) and indicated an important role of the nanoscale arrangement of gephyrin and the associated receptors at post-synaptic sites (Groeneweg et al., 2018; Specht, 2019). Further functional and *in-silico* studies (Pennacchietti et al., 2017) confirmed that gephyrin organizes the receptors in distinct nanoscale structures, which shape GABAergic synaptic potentiation and reduce current variability. The stability, oligomerization and receptor binding of gephyrin are highly regulated and exert tight control over receptor numbers at post-synaptic sites, affecting synaptic strength (Alvarez, 2017; Groeneweg et al., 2018). Biophysical (Maric et al., 2011) and structural (Maric et al., 2014, 2015) studies provided first insights into the structure and function of the gephyrin-GABA_A_R complexes and demonstrated that at least the GABA_A_R α1–3 and GlyR β subunits bind to an overlapping site within gephyrin in a mutually exclusive fashion (Maric et al., 2011, 2014; Figure 1 and Table 1). Microscopy studies substantiated that gephyrin acts as a dynamic post-synaptic platform for both, GABA_A_Rs and GlyRs (Specht et al., 2013), and that receptor residence times at the postsynapse depend on binding affinities and distinct post-translational modifications (Mukherjee et al., 2011; Specht et al., 2011). The concept of ligand competition for gephyrin binding, therefore, lends an elegant explanation for the comparably high diffusion dynamics of high-affinity gephyrin binding receptors. This phenomenon may be the natural solution to the biological requirement to maintain distinct subsets of receptor subtypes in high density at post-synaptic sites, while at the same time allowing for the rapid exchange of these receptors and scalability through diffusion dynamics (Specht, 2019). This model is also consistent with the observation of reciprocal stabilization of receptors, and the underlying protein scaffold, at inhibitory synapses (Essrich et al., 1998).

Gephyrin itself is dynamically regulated, affecting GABA_A_R diffusion and contributing to input-specific adaptations at postsynaptic sites (Chen et al., 2012; Villa et al., 2016; Battaglia et al., 2018). Gephyrin phosphorylation at Ser268 and Ser270, located in the intrinsically disordered central region of the protein, directly affects GABAergic signaling (Tyagarajan et al., 2011b, 2013) and induce gephyrin-mediated remodeling of GABAergic synapses in specific neuronal cell-types (Flores et al., 2015). Despite its major functional relevance only a few of the molecular interfaces that engage with the central region of gephyrin could be identified (Groeneweg et al., 2018). The underlying molecular mechanisms for these gephyrin phosphorylation-induced GABA_A_R synapse dynamics remain to be explored in a comprehensive approach that includes an extensive alternative splicing and complex post-translational modification patterns of this region. Identification of the targeted binding pockets and insights into the binding affinities of the modified and unmodified peptide regions within the central region of gephyrin could shed light on the enigmatic molecular mechanisms of gephyrin multimerization, degradation and the tuning of its ligand binding affinities. Additionally, gephyrin isoforms are tissue-specific (Paarmann et al., 2006), therefore, molecules targeting distinct gephyrin splice variants may display pronounced effects in distinct tissues or brain regions.

## Targeting the Gephyrin-GABA_A_R Complex

Gephyrin’s crucial role in glycinergic and GABAergic transmission made it a major pharmacological target. The modulation of synaptic responses *via* gephyrin was achieved more than a decade ago using intrabodies (Zacchi et al., 2008), and a related approach turned out to be useful for acutely removing inhibitory synapses (Gross et al., 2016). Since then, several studies made an impressive progress in the development of agents affecting the intracellular interplay of GABA_A_Rs. One such example is artemisinins [Figure Figure 2B(10)]. Li et al. (2017) found that artemisinins, lactones derived from the Qinghao plant, affect pancreatic cells by binding gephyrin and modifying GABA_A_R signaling. Kasaragod et al. (2019) identified the artemisinin binding site within gephyrin and showed that application of artemisinins reduces gephyrin and GABA_A_Rs clustering, making artemisinins the first small molecule lead compounds for a new class of inhibitory neurotransmission modulators. Strikingly, the druggable artemisinin-binding pocket overlaps with the universal receptor binding region of gephyrin, which is critical for the interaction with GABA_A_ and glycine receptors (Kasaragod et al., 2019). Thermodynamic and structural studies (Maric et al., 2011, 2014) identified the “hotspot” fragments of GABA_A_ and glycine receptors that bind to gephyrin. Biomimetic optimization of the “hotspots” amino acid sequence, enhanced the affinity of the resulting peptide ligands 46,000-fold compared to the corresponding native peptides (Maric et al., 2015, 2017; Figures 2B,C). Further *in vitro* applications of these new super binder peptide reduced GABA_A_R α2 conductivity and clustering, providing evidence that GABA_A_R-associated proteins can be successfully targeted with modified peptides to modulate fast synaptic inhibition (Maric et al., 2017).

## Targeting Non-scaffold GABA_A_R Associated Proteins

GABA_A_R trafficking is pivotal for the plasticity (Luscher et al., 2011) and the development (Lorenz-Guertin and Jacob, 2018) of inhibitory synapses, consequently, dysfunction of the GABA_A_R cycling is involved in various neurological disorders (Smith and Kittler, 2010; Mele et al., 2019). Noteworthy, phosphorylation of the intracellular GABA_A_R sites, that are involved in the trafficking of the receptors, has been identified to control receptor numbers and their concentration at synaptic sites (Comenencia-Ortiz et al., 2014; Nakamura et al., 2015), a mechanism that proves to be critical for the physiological function of inhibitory synapses (Vien et al., 2015). Therefore, targeting protein-protein interactions (PPIs) that mediate GABA_A_R trafficking, endocytosis, degradation or recycling, is a promising pharmacological strategy. The proposed direct protein interactors are numerous, among them are muskelin (Heisler et al., 2011), GABARAP (Wang et al., 1999), the brefeldin-A inhibited GDP/GTP exchange factor 2 (Charych et al., 2004), phospholipase C-related catalytically inactive proteins 1 and 2 (Mizokami et al., 2007), N-ethylmaleimide sensitive factor (Goto et al., 2005), neurobeachin (Nair et al., 2013), Huntingtin-associated protein 1, calcium-modulating cyclophilin ligand (Kittler et al., 2004; Yuan et al., 2008) and the clathrin adaptor protein AP2 (Kittler et al., 2005).

The AP2-GABA_A_R interaction rapidly modulates synaptic GABA_A_R numbers, inhibitory synaptic strength, neuronal excitability, and notably, affects animal behavior (Kittler et al., 2000, 2005, 2008; Tretter et al., 2009). The μ2 subunit of the clathrin adaptor protein AP2 binds with high affinity to linear and short peptide motifs within the intracellular regions of specific GABA_A_R subunits (Table 1). Short GABA_A_R derived peptides, that effectively compete with AP2 binding, were successfully used to block the receptor internalization in hippocampal neurons, increasing surface concentration of GABA_A_R clusters by 50% (Smith et al., 2012) and enhancing the strength of inhibitory synapses (Kittler et al., 2008). AP2 antagonists demonstrate that the modulation of GABA_A_R interactions with its intracellular trafficking partners is an alternative way to influence GABAergic signaling.

## Perspectives

Ongoing research uncovered original, seemingly contrasting, strategies of GABAergic signaling modulation. On the one hand, ligands disrupting gephyrin-GABA_A_R clustering, like artemisinins or “super binding peptides,” could reduce the GABA_A_R synaptic concentration and function. On the other hand, peptides hampering receptor interaction with AP2 trafficking protein increased the synaptic receptor levels. In theory, these approaches could be applied together to achieve bi-directional modulation of inhibitory neurotransmission, promoting a shift in the dynamic equilibrium from phasic to tonic neuronal response.

Those new strategies of GABAergic neurotransmission modulation possess an untapped clinical potential. Agents targeting GABA_A_R associated scaffold or trafficking proteins could be applied wherever abnormal GABAergic activity or regulation is involved in pathogenesis. In status epilepticus patients develop a time-dependent pharmacoresistance to GABAergic agents, probably, due to GABA_A_R internalization (Naylor et al., 2005). In benzodiazepine tolerance linked to prolonged benzodiazepine use, neurons continuously exposed to diazepam lose postsynaptic GABA_A_Rs (Nicholson et al., 2018). Both pathologies are related to the reduction of available postsynaptic GABA_A_Rs and both could potentially be alleviated by targeting GABA_A_R-associated proteins. Stabilization of the gephyrin-receptor scaffolds at inhibitory postsynapses with molecules that mimic the stabilizing action of CB (Saiepour et al., 2010) could help prevent GABA_A_R loss and preserve inhibitory neurotransmission, alternatively, applying AP2 inhibitors could reduce GABA_A_R internalization and reverse the loss of postsynaptic GABA_A_Rs. Those examples illustrate the potential of GABAergic modulators as adjuvants ameliorating the effect of existing potent drugs, whereas in epilepsy or other diseases involving deregulation of inhibitory neurotransmission they could be applied as stand-alone therapeutics.

We expect that the study of GABA_A_R intracellular interactors, accelerated by *in-silico* predictions and high throughput approaches, will lead to the discovery of novel GABAergic modulators. Affinity, selectivity, bioavailability and immunogenicity of these compounds would have to be optimized for clinical applications, where peptide-based ligands could be further evolved by the introduction of unnatural amino acids, cyclization and other chemical modifications.

Microscopy is an additional intriguing application of these molecules. The enhanced affinity and specificity of the engineered peptide-based compounds allowed to pioneer their use as fluorescent probes [Figure Figure 2B(5)], enabling live neuronal staining and visualization of inhibitory post synaptic sites with submicron resolution (Maric et al., 2017). Compact fluorescent peptides, developed from these super binding peptides, bring several advantages over conventional staining agents, namely the antibodies. In contrast to antibodies, peptide probes are live cell compatible and could provide better resolution and localization precision, since the fluorophore, owing to its small size, stays close to the target surface, reducing the linkage error. Moreover, highly affine and selective peptides could achieve stoichiometric labeling, enabling quantification of the target protein.

Here, we discussed how the targeting of GABA_A_R associated proteins could prove to be a versatile pharmacological strategy with clinical potential. Further, we suggested that when combined with state-of-the-art super-resolution microscopy methods, the peptide-based fluorescent probes may resolve the nanoscale architecture of synapses in unprecedented detail. We anticipate that the discovery of additional GABA_A_R interactors could open the way for the development of new imaging tools and alternative pharmacological approaches.

## Author Contributions

VK and HM wrote the manuscript and prepared the figures.

## Conflict of Interest Statement

The authors declare that the research was conducted in the absence of any commercial or financial relationships that could be construed as a potential conflict of interest.

## References

[B1] Ali Rodriguez R. Joya C. Hines R. M. (2018). Common ribs of inhibitory synaptic dysfunction in the umbrella of neurodevelopmental disorders. Front. Mol. Neurosci. 11:132. 10.3389/fnmol.2018.00132 29740280 PMC5928253

[B2] Alvarez F. J. (2017). Gephyrin and the regulation of synaptic strength and dynamics at glycinergic inhibitory synapses. Brain Res. Bull. 129, 50–65. 10.1016/j.brainresbull.2016.09.003 27612963

[B3] Bach A. Clausen B. H. Moller M. Vestergaard B. Chi C. N. Round A.. (2012). A high-affinity, dimeric inhibitor of PSD-95 bivalently interacts with PDZ1–2 and protects against ischemic brain damage. Proc. Natl. Acad. Sci. U S A 109, 3317–3322. 10.1073/pnas.1113761109 22343531 PMC3295328

[B4] Bach A. Eildal J. N. Stuhr-Hansen N. Deeskamp R. Gottschalk M. Pedersen S. W.. (2011). Cell-permeable and plasma-stable peptidomimetic inhibitors of the postsynaptic density-95/N-methyl-D-aspartate receptor interaction. J. Med. Chem. 54, 1333–1346. 10.1021/jm1013924 21322614

[B5] Battaglia S. Renner M. Russeau M. Come E. Tyagarajan S. K. Lévi S. (2018). Activity-dependent inhibitory synapse scaling is determined by gephyrin phosphorylation and subsequent regulation of GABA_A_ receptor diffusion. eNeuro 5:ENEURO.0203-17.2017. 10.1523/eneuro.0203-17.2017 29379879 PMC5780843

[B6] Brady M. L. Jacob T. C. (2015). Synaptic localization of α5 GABA_A_ receptors *via* gephyrin interaction regulates dendritic outgrowth and spine maturation. Dev. Neurobiol. 75, 1241–1251. 10.1002/dneu.22280 25663431 PMC5240477

[B7] Charych E. I. Yu W. Li R. Serwanski D. R. Miralles C. P. Li X.. (2004). A four PDZ domain-containing splice variant form of GRIP1 is localized in GABAergic and glutamatergic synapses in the brain. J. Biol. Chem. 279, 38978–38990. 10.1074/jbc.m405786200 15226318

[B8] Chen J. L. Villa K. L. Cha J. W. So P. T. Kubota Y. Nedivi E. (2012). Clustered dynamics of inhibitory synapses and dendritic spines in the adult neocortex. Neuron 74, 361–373. 10.1016/j.neuron.2012.02.030 22542188 PMC3340582

[B9] Chiou T.-T. Long P. Schumann-Gillett A. Kanamarlapudi V. Haas S. A. Harvey K.. (2019). Mutation p.R356Q in the collybistin phosphoinositide binding site is associated with mild intellectual disability. Front. Mol. Neurosci. 12:60. 10.3389/fnmol.2019.00060 30914922 PMC6422930

[B10] Choii G. Ko J. (2015). Gephyrin: a central GABAergic synapse organizer. Exp. Mol. Med. 47:e158. 10.1038/emm.2015.5 25882190

[B11] Comenencia-Ortiz E. Moss S. J. Davies P. A. (2014). Phosphorylation of GABA_A_ receptors influences receptor trafficking and neurosteroid actions. Psychopharmacology 231, 3453–3465. 10.1007/s00213-014-3617-z 24847959 PMC4135009

[B12] Darmani G. Zipser C. M. Bohmer G. M. Deschet K. Muller-Dahlhaus F. Belardinelli P.. (2016). Effects of the selective α5-GABAAR antagonist S44819 on excitability in the human brain: a TMS-EMG and TMS-EEG phase I study. J. Neurosci. 36, 12312–12320. 10.1523/JNEUROSCI.1689-16.2016 27927951 PMC6601976

[B13] Davenport E. C. Pendolino V. Kontou G. Mcgee T. P. Sheehan D. F. López-Doménech G.. (2017). An essential role for the tetraspanin LHFPL4 in the cell-type-specific targeting and clustering of synaptic GABA_A_ receptors. Cell Rep. 21, 70–83. 10.1016/j.celrep.2017.09.025 28978485 PMC5640807

[B14] de Jonge J. C. Vinkers C. H. Hulshoff Pol H. E. Marsman A. (2017). GABAergic mechanisms in schizophrenia: linking postmortem and *in vivo* studies. Front. Psychiatry 8:118. 10.3389/fpsyt.2017.00118 28848455 PMC5554536

[B15] Essrich C. Lorez M. Benson J. A. Fritschy J. M. Luscher B. (1998). Postsynaptic clustering of major GABA_A_ receptor subtypes requires the γ 2 subunit and gephyrin. Nat. Neurosci. 1, 563–571. 10.1038/2798 10196563

[B16] Feng G. Tintrup H. Kirsch J. Nichol M. C. Kuhse J. Betz H.. (1998). Dual requirement for gephyrin in glycine receptor clustering and molybdoenzyme activity. Science 282, 1321–1324. 10.1126/science.282.5392.1321 9812897

[B17] Flores C. E. Nikonenko I. Mendez P. Fritschy J. M. Tyagarajan S. K. Muller D. (2015). Activity-dependent inhibitory synapse remodeling through gephyrin phosphorylation. Proc. Natl. Acad. Sci. U S A 112, E65–E72. 10.1073/pnas.1411170112 25535349 PMC4291629

[B18] Fritschy J. M. Panzanelli P. (2014). GABA receptors and plasticity of inhibitory neurotransmission in the central nervous system. Eur. J. Neurosci. 39, 1845–1865. 10.1111/ejn.12534 24628861

[B19] Goto H. Terunuma M. Kanematsu T. Misumi Y. Moss S. J. Hirata M. (2005). Direct interaction of N-ethylmaleimide-sensitive factor with GABA_A_ receptor β subunits. Mol. Cell. Neurosci. 30, 197–206. 10.1016/j.mcn.2005.07.006 16095914

[B20] Govindpani K. Calvo-Flores Guzman B. Vinnakota C. Waldvogel H. J. Faull R. L. Kwakowsky A. (2017). Towards a better understanding of GABAergic remodeling in Alzheimer’s disease. Int. J. Mol. Sci. 18:E1813. 10.3390/ijms18081813 28825683 PMC5578199

[B21] Groeneweg F. L. Trattnig C. Kuhse J. Nawrotzki R. A. Kirsch J. (2018). Gephyrin: a key regulatory protein of inhibitory synapses and beyond. Histochem. Cell Biol. 150, 489–508. 10.1007/s00418-018-1725-2 30264265

[B22] Gross G. G. Straub C. Perez-Sanchez J. Dempsey W. P. Junge J. A. Roberts R. W.. (2016). An E3-ligase-based method for ablating inhibitory synapses. Nat. Methods 13, 673–678. 10.1038/nmeth.3894 27271196 PMC5312699

[B23] Hammond M. C. Harris B. Z. Lim W. A. Bartlett P. A. (2006). β strand peptidomimetics as potent PDZ domain ligands. Chem. Biol. 13, 1247–1251. 10.1016/j.chembiol.2006.11.010 17185220

[B24] Harvey K. Duguid I. C. Alldred M. J. Beatty S. E. Ward H. Keep N. H.. (2004). The GDP-GTP exchange factor collybistin: an essential determinant of neuronal gephyrin clustering. J. Neurosci. 24, 5816–5826. 10.1523/JNEUROSCI.1184-04.2004 15215304 PMC6729214

[B25] Hausrat T. J. Muhia M. Gerrow K. Thomas P. Hirdes W. Tsukita S.. (2015). Radixin regulates synaptic GABA_A_ receptor density and is essential for reversal learning and short-term memory. Nat. Commun. 6:6872. 10.1038/ncomms7872 25891999 PMC4411296

[B26] Heisler F. F. Loebrich S. Pechmann Y. Maier N. Zivkovic A. R. Tokito M.. (2011). Muskelin regulates actin filament- and microtubule-based GABA_A_ receptor transport in neurons. Neuron 70, 66–81. 10.1016/j.neuron.2011.03.008 21482357 PMC3101366

[B27] Hines R. M. Maric H. M. Hines D. J. Modgil A. Panzanelli P. Nakamura Y.. (2018). Developmental seizures and mortality result from reducing GABA_A_ receptor α2-subunit interaction with collybistin. Nat. Commun. 9:3130. 10.1038/s41467-018-05481-1 30087324 PMC6081406

[B28] Kang Y. Ge Y. Cassidy R. M. Lam V. Luo L. Moon K. M.. (2014). A combined transgenic proteomic analysis and regulated trafficking of neuroligin-2. J. Biol. Chem. 289, 29350–29364. 10.1074/jbc.m114.549279 25190809 PMC4200284

[B30] Kasaragod V. B. Hausrat T. J. Schaefer N. Kuhn M. Christensen N. R. Tessmer I.. (2019). Elucidating the molecular basis for inhibitory neurotransmission regulation by artemisinins. Neuron 101, 673.e11–689.e11. 10.1016/j.neuron.2019.01.001 30704910

[B29] Kasaragod V. B. Schindelin H. (2018). Structure-function relationships of glycine and GABA_A_ receptors and their interplay with the scaffolding protein gephyrin. Front. Mol. Neurosci. 11:317. 10.3389/fnmol.2018.00317 30258351 PMC6143783

[B31] Kawaguchi K. Yoshida S. Hatano R. Asano S. (2017). Pathophysiological roles of ezrin/radixin/moesin proteins. Biol. Pharm. Bull. 40, 381–390. 10.1248/bpb.b16-01011 28381792

[B32] Kins S. Betz H. Kirsch J. (2000). Collybistin, a newly identified brain-specific GEF, induces submembrane clustering of gephyrin. Nat. Neurosci. 3, 22–29. 10.1038/71096 10607391

[B33] Kitano K. Yusa F. Hakoshima T. (2006). Structure of dimerized radixin FERM domain suggests a novel masking motif in C-terminal residues 295-304. Acta Crystallogr. Sect. F Struct. Biol. Cryst. Commun. 62, 340–345. 10.1107/s1744309106010062 16582480 PMC2222584

[B34] Kittler J. T. Arancibia-Carcamo I. L. Moss S. J. (2004). Association of GRIP1 with a GABA_A_ receptor associated protein suggests a role for GRIP1 at inhibitory synapses. Biochem. Pharmacol. 68, 1649–1654. 10.1016/j.bcp.2004.07.028 15451408

[B35] Kittler J. T. Chen G. Honing S. Bogdanov Y. Mcainsh K. Arancibia-Carcamo I. L.. (2005). Phospho-dependent binding of the clathrin AP2 adaptor complex to GABA_A_ receptors regulates the efficacy of inhibitory synaptic transmission. Proc. Natl. Acad. Sci. U S A 102, 14871–14876. 10.1073/pnas.0506653102 16192353 PMC1253579

[B36] Kittler J. T. Chen G. Kukhtina V. Vahedi-Faridi A. Gu Z. Tretter V.. (2008). Regulation of synaptic inhibition by phospho-dependent binding of the AP2 complex to a YECL motif in the GABA_A_ receptor γ2 subunit. Proc. Natl. Acad. Sci. U S A 105, 3616–3621. 10.1073/pnas.0707920105 18305175 PMC2265186

[B37] Kittler J. T. Delmas P. Jovanovic J. N. Brown D. A. Smart T. G. Moss S. J. (2000). Constitutive endocytosis of GABA_A_ receptors by an association with the adaptin AP2 complex modulates inhibitory synaptic currents in hippocampal neurons. J. Neurosci. 20, 7972–7977. 10.1523/jneurosci.20-21-07972.2000 11050117 PMC6772725

[B38] Kneussel M. Brandstatter J. H. Laube B. Stahl S. Muller U. Betz H. (1999). Loss of postsynaptic GABA_A_ receptor clustering in gephyrin-deficient mice. J. Neurosci. 19, 9289–9297. 10.1523/jneurosci.19-21-09289.1999 10531433 PMC6782938

[B39] Kowalczyk S. Winkelmann A. Smolinsky B. Forstera B. Neundorf I. Schwarz G.. (2013). Direct binding of GABA_A_ receptor β2 and β3 subunits to gephyrin. Eur. J. Neurosci. 37, 544–554. 10.1111/ejn.12078 23205938

[B40] Krueger-Burg D. Papadopoulos T. Brose N. (2017). Organizers of inhibitory synapses come of age. Curr. Opin. Neurobiol. 45, 66–77. 10.1016/j.conb.2017.04.003 28460365

[B41] Laverty D. Desai R. Uchański T. Masiulis S. Stec W. J. Malinauskas T.. (2019). Cryo-EM structure of the human α1β3γ2 GABA_A_ receptor in a lipid bilayer. Nature 565, 516–520. 10.1038/s41586-018-0833-4 30602789 PMC6364807

[B42] Lee V. Maguire J. (2014). The impact of tonic GABA_A_ receptor-mediated inhibition on neuronal excitability varies across brain region and cell type. Front. Neural Circuits 8:3. 10.3389/fncir.2014.00003 24550784 PMC3909947

[B43] Letellier M. Sziber Z. Chamma I. Saphy C. Papasideri I. Tessier B.. (2018). A unique intracellular tyrosine in neuroligin-1 regulates AMPA receptor recruitment during synapse differentiation and potentiation. Nat. Commun. 9:3979. 10.1038/s41467-018-06220-2 30266896 PMC6162332

[B44] Li J. Casteels T. Frogne T. Ingvorsen C. Honore C. Courtney M.. (2017). Artemisinins target GABA_A_ receptor signaling and impair α cell identity. Cell 168, 86.e15–100.e15. 10.1016/j.cell.2016.11.010 27916275 PMC5236063

[B45] Loebrich S. Bahring R. Katsuno T. Tsukita S. Kneussel M. (2006). Activated radixin is essential for GABA_A_ receptor α5 subunit anchoring at the actin cytoskeleton. EMBO J. 25, 987–999. 10.1038/sj.emboj.7600995 16467845 PMC1409722

[B46] Long P. May M. M. James V. M. Granno S. Johnson J. P. Tarpey P.. (2016). Missense mutation R338W in ARHGEF9 in a family with X-linked intellectual disability with variable macrocephaly and macro-orchidism. Front. Mol. Neurosci. 8:83. 10.3389/fnmol.2015.00083 26834553 PMC4719118

[B47] Lorenz-Guertin J. M. Jacob T. C. (2018). GABA type a receptor trafficking and the architecture of synaptic inhibition. Dev. Neurobiol. 78, 238–270. 10.1002/dneu.22536 28901728 PMC6589839

[B48] Löscher W. Rogawski M. A. (2012). How theories evolved concerning the mechanism of action of barbiturates. Epilepsia 53, 12–25. 10.1111/epi.12025 23205959

[B49] Lu W. Bromley-Coolidge S. Li J. (2017). Regulation of GABAergic synapse development by postsynaptic membrane proteins. Brain Res. Bull. 129, 30–42. 10.1016/j.brainresbull.2016.07.004 27453545 PMC5253122

[B50] Ludolphs M. Schneeberger D. Soykan T. Schafer J. Papadopoulos T. Brose N.. (2016). Specificity of collybistin-phosphoinositide interactions: impact of the individual protein domains. J. Biol. Chem. 291, 244–254. 10.1074/jbc.m115.673400 26546675 PMC4697159

[B51] Luscher B. Fuchs T. Kilpatrick C. L. (2011). GABA_A_ receptor trafficking-mediated plasticity of inhibitory synapses. Neuron 70, 385–409. 10.1016/j.neuron.2011.03.024 21555068 PMC3093971

[B52] Maher M. P. Matta J. A. Gu S. Seierstad M. Bredt D. S. (2017). Getting a handle on neuropharmacology by targeting receptor-associated proteins. Neuron 96, 989–1001. 10.1016/j.neuron.2017.10.001 29216460

[B53] Maric H. M. Hausrat T. J. Neubert F. Dalby N. O. Doose S. Sauer M.. (2017). Gephyrin-binding peptides visualize postsynaptic sites and modulate neurotransmission. Nat. Chem. Biol. 13, 153–160. 10.1038/nchembio.2246 27893705

[B54] Maric H. M. Kasaragod V. B. Haugaard-Kedstrom L. Hausrat T. J. Kneussel M. Schindelin H.. (2015). Design and synthesis of high-affinity dimeric inhibitors targeting the interactions between gephyrin and inhibitory neurotransmitter receptors. Angew. Chem. Int. Ed Engl. 54, 490–494. 10.1002/anie.201409043 25413248

[B55] Maric H. M. Kasaragod V. B. Hausrat T. J. Kneussel M. Tretter V. Stromgaard K.. (2014). Molecular basis of the alternative recruitment of GABA_A_ versus glycine receptors through gephyrin. Nat. Commun. 5:5767. 10.1038/ncomms6767 25531214

[B56] Maric H. M. Mukherjee J. Tretter V. Moss S. J. Schindelin H. (2011). Gephyrin-mediated γ-aminobutyric acid type A and glycine receptor clustering relies on a common binding site. J. Biol. Chem. 286, 42105–42114. 10.1074/jbc.m111.303412 22006921 PMC3234978

[B57] Masiulis S. Desai R. Uchański T. Serna Martin I. Laverty D. Karia D.. (2019). GABA_A_ receptor signalling mechanisms revealed by structural pharmacology. Nature 565, 454–459. 10.1038/s41586-018-0832-5 30602790 PMC6370056

[B58] Mayer S. Kumar R. Jaiswal M. Soykan T. Ahmadian M. R. Brose N.. (2013). Collybistin activation by GTP-TC10 enhances postsynaptic gephyrin clustering and hippocampal GABAergic neurotransmission. Proc. Natl. Acad. Sci. U S A 110, 20795–20800. 10.1073/pnas.1309078110 24297911 PMC3870750

[B59] Mele M. Costa R. O. Duarte C. B. (2019). Alterations in GABA_A_-receptor trafficking and synaptic dysfunction in brain disorders. Front. Cell. Neurosci. 13:77. 10.3389/fncel.2019.00077 30899215 PMC6416223

[B60] Miller P. S. Aricescu A. R. (2014). Crystal structure of a human GABA_A_ receptor. Nature 512, 270–275. 10.1038/nature13293 24909990 PMC4167603

[B61] Miller P. S. Scott S. Masiulis S. De Colibus L. Pardon E. Steyaert J.. (2017). Structural basis for GABA_A_ receptor potentiation by neurosteroids. Nat. Struct. Mol. Biol. 24, 986–992. 10.1038/nsmb.3484 28991263 PMC6166781

[B62] Mizokami A. Kanematsu T. Ishibashi H. Yamaguchi T. Tanida I. Takenaka K.. (2007). Phospholipase C-related inactive protein is involved in trafficking of γ2 subunit-containing GABA_A_ receptors to the cell surface. J. Neurosci. 27, 1692–1701. 10.1523/jneurosci.3155-06.2007 17301177 PMC6673751

[B63] Mody I. (2019). GABA_A_R modulator for postpartum depression. Cell 176:1. 10.1016/j.cell.2018.12.016 30633900

[B64] Mody I. Pearce R. A. (2004). Diversity of inhibitory neurotransmission through GABA_A_ receptors. Trends Neurosci. 27, 569–575. 10.1016/j.tins.2004.07.002 15331240

[B65] Mukherjee J. Kretschmannova K. Gouzer G. Maric H. M. Ramsden S. Tretter V.. (2011). The residence time of GABA_A_Rs at inhibitory synapses is determined by direct binding of the receptor α1 subunit to gephyrin. J. Neurosci. 31, 14677–14687. 10.1523/jneurosci.2001-11.2011 21994384 PMC3202462

[B66] Nair R. Lauks J. Jung S. Cooke N. E. De Wit H. Brose N.. (2013). Neurobeachin regulates neurotransmitter receptor trafficking to synapses. J. Cell Biol. 200, 61–80. 10.1083/jcb.201207113 23277425 PMC3542797

[B67] Nakamura Y. Darnieder L. M. Deeb T. Z. Moss S. J. (2015). Regulation of GABA_A_Rs by phosphorylation. Adv. Pharmacol. 72, 97–146. 10.1016/bs.apha.2014.11.008 25600368 PMC5337123

[B68] Nakamura Y. Morrow D. H. Modgil A. Huyghe D. Deeb T. Z. Lumb M. J.. (2016). Proteomic characterization of inhibitory synapses using a novel pHluorin-tagged γ-aminobutyric acid receptor, type A (GABA_A_), α2 subunit knock-in mouse. J. Biol. Chem. 291, 12394–12407. 10.1074/jbc.m116.724443 27044742 PMC4933285

[B69] Naylor D. E. Liu H. Wasterlain C. G. (2005). Trafficking of GABA_A_ receptors, loss of inhibition, and a mechanism for pharmacoresistance in status epilepticus. J. Neurosci. 25, 7724–7733. 10.1523/jneurosci.4944-04.2005 16120773 PMC6725248

[B70] Nguyen Q. A. Horn M. E. Nicoll R. A. (2016). Distinct roles for extracellular and intracellular domains in neuroligin function at inhibitory synapses. Elife 5:e19236. 10.7554/elife.19236 27805570 PMC5098909

[B71] Nicholson M. W. Sweeney A. Pekle E. Alam S. Ali A. B. Duchen M.. (2018). Diazepam-induced loss of inhibitory synapses mediated by PLCdelta/ Ca^2+^/calcineurin signalling downstream of GABA_A_ receptors. Mol. Psychiatry 23, 1851–1867. 10.1038/s41380-018-0100-y 29904150 PMC6232101

[B72] Olsen R. W. (2018). GABA_A_ receptor: Positive and negative allosteric modulators. Neuropharmacology 136, 10–22. 10.1016/j.neuropharm.2018.01.036 29407219 PMC6027637

[B73] Olsen R. W. Sieghart W. (2009). GABA A receptors: subtypes provide diversity of function and pharmacology. Neuropharmacology 56, 141–148. 10.1016/j.neuropharm.2008.07.045 18760291 PMC3525320

[B74] Paarmann I. Schmitt B. Meyer B. Karas M. Betz H. (2006). Mass spectrometric analysis of glycine receptor-associated gephyrin splice variants. J. Biol. Chem. 281, 34918–34925. 10.1074/jbc.m607764200 17001074

[B75] Palma E. Ruffolo G. Cifelli P. Roseti C. Vliet E. A. V. Aronica E. (2017). Modulation of GABA_A_ receptors in the treatment of epilepsy. Curr. Pharm. Des. 23, 5563–5568. 10.2174/1381612823666170809100230 28799512

[B76] Papadopoulos T. Eulenburg V. Reddy-Alla S. Mansuy I. M. Li Y. Betz H. (2008). Collybistin is required for both the formation and maintenance of GABAergic postsynapses in the hippocampus. Mol. Cell. Neurosci. 39, 161–169. 10.1016/j.mcn.2008.06.006 18625319

[B77] Papadopoulos T. Korte M. Eulenburg V. Kubota H. Retiounskaia M. Harvey R. J.. (2007). Impaired GABAergic transmission and altered hippocampal synaptic plasticity in collybistin-deficient mice. EMBO J. 26, 3888–3899. 10.1038/sj.emboj.7601819 17690689 PMC1994120

[B78] Pennacchietti F. Vascon S. Nieus T. Rosillo C. Das S. Tyagarajan S. K.. (2017). Nanoscale molecular reorganization of the inhibitory postsynaptic density is a determinant of GABAergic synaptic potentiation. J. Neurosci. 37, 1747–1756. 10.1523/JNEUROSCI.0514-16.2016 28073939 PMC6589977

[B79] Pettem K. L. Yokomaku D. Takahashi H. Ge Y. Craig A. M. (2013). Interaction between autism-linked MDGAs and neuroligins suppresses inhibitory synapse development. J. Cell Biol. 200, 321–336. 10.1083/jcb.201206028 23358245 PMC3563690

[B80] Pfeiffer F. Graham D. Betz H. (1982). Purification by affinity chromatography of the glycine receptor of rat spinal cord. J. Biol. Chem. 257, 9389–9393. 6286620

[B81] Poulopoulos A. Aramuni G. Meyer G. Soykan T. Hoon M. Papadopoulos T.. (2009). Neuroligin 2 drives postsynaptic assembly at perisomatic inhibitory synapses through gephyrin and collybistin. Neuron 63, 628–642. 10.1016/j.neuron.2009.08.023 19755106

[B82] Reddy-Alla S. Schmitt B. Birkenfeld J. Eulenburg V. Dutertre S. Bohringer C.. (2010). PH-domain-driven targeting of collybistin but not Cdc42 activation is required for synaptic gephyrin clustering. Eur. J. Neurosci. 31, 1173–1184. 10.1111/j.1460-9568.2010.07149.x 20345913

[B83] Saiepour L. Fuchs C. Patrizi A. Sassoè-Pognetto M. Harvey R. J. Harvey K. (2010). Complex role of collybistin and gephyrin in GABA_A_ receptor clustering. J. Biol. Chem. 285, 29623–29631. 10.1074/jbc.M110.121368 20622020 PMC2937993

[B84] Sainlos M. Tigaret C. Poujol C. Olivier N. B. Bard L. Breillat C.. (2011). Biomimetic divalent ligands for the acute disruption of synaptic AMPAR stabilization. Nat. Chem. Biol. 7, 81–91. 10.1038/nchembio.498 21186349

[B85] Sancar F. Czajkowski C. (2011). Allosteric modulators induce distinct movements at the GABA-binding site interface of the GABA-A receptor. Neuropharmacology 60, 520–528. 10.1016/j.neuropharm.2010.11.009 21093460 PMC3026633

[B86] Smith K. R. Kittler J. T. (2010). The cell biology of synaptic inhibition in health and disease. Curr. Opin. Neurobiol. 20, 550–556. 10.1016/j.conb.2010.06.001 20650630

[B87] Smith K. R. Muir J. Rao Y. Browarski M. Gruenig M. C. Sheehan D. F.. (2012). Stabilization of GABA_A_ receptors at endocytic zones is mediated by an AP2 binding motif within the GABA_A_ receptor β3 subunit. J. Neurosci. 32, 2485–2498. 10.1523/JNEUROSCI.1622-11.2011 22396422 PMC6621817

[B88] Soykan T. Schneeberger D. Tria G. Buechner C. Bader N. Svergun D.. (2014). A conformational switch in collybistin determines the differentiation of inhibitory postsynapses. EMBO J. 33, 2113–2133. 10.15252/embj.201488143 25082542 PMC4195776

[B89] Specht C. G. (2019). Fractional occupancy of synaptic binding sites and the molecular plasticity of inhibitory synapses. Neuropharmacology [Epub ahead of print]. 10.1016/j.neuropharm.2019.01.008 30648560

[B90] Specht C. G. Grunewald N. Pascual O. Rostgaard N. Schwarz G. Triller A. (2011). Regulation of glycine receptor diffusion properties and gephyrin interactions by protein kinase C. EMBO J. 30, 3842–3853. 10.1038/emboj.2011.276 21829170 PMC3173796

[B91] Specht C. G. Izeddin I. Rodriguez P. C. El Beheiry M. Rostaing P. Darzacq X.. (2013). Quantitative nanoscopy of inhibitory synapses: counting gephyrin molecules and receptor binding sites. Neuron 79, 308–321. 10.1016/j.neuron.2013.05.013 23889935

[B92] Takai Y. Kitano K. Terawaki S. Maesaki R. Hakoshima T. (2007). Crystallographic characterization of the radixin FERM domain bound to the cytoplasmic tails of adhesion molecules CD43 and PSGL-1. Acta Crystallogr. Sect. F Struct. Biol. Cryst. Commun. 63, 49–51. 10.1107/s1744309106054145 17183174 PMC2330108

[B94] Terawaki S. Kitano K. Aoyama M. Hakoshima T. (2008). Crystallographic characterization of the radixin FERM domain bound to the cytoplasmic tail of membrane-type 1 matrix metalloproteinase (MT1-MMP). Acta Crystallogr. Sect. F Struct. Biol. Cryst. Commun. 64, 911–913. 10.1107/s1744309108026869 18931433 PMC2564885

[B93] Terawaki S. Kitano K. Hakoshima T. (2007). Structural basis for type II membrane protein binding by ERM proteins revealed by the radixin-neutral endopeptidase 24.11 (NEP) complex. J. Biol. Chem. 282, 19854–19862. 10.1074/jbc.m609232200 17459884

[B95] Tretter V. Jacob T. C. Mukherjee J. Fritschy J. M. Pangalos M. N. Moss S. J. (2008). The clustering of GABA_A_ receptor subtypes at inhibitory synapses is facilitated *via* the direct binding of receptor α 2 subunits to gephyrin. J. Neurosci. 28, 1356–1365. 10.1523/JNEUROSCI.5050-07.2008 18256255 PMC6671568

[B96] Tretter V. Kerschner B. Milenkovic I. Ramsden S. L. Ramerstorfer J. Saiepour L.. (2011). Molecular basis of the γ-aminobutyric acid A receptor α3 subunit interaction with the clustering protein gephyrin. J. Biol. Chem. 286, 37702–37711. 10.1074/jbc.m111.291336 21880742 PMC3199513

[B97] Tretter V. Mukherjee J. Maric H. M. Schindelin H. Sieghart W. Moss S. J. (2012). Gephyrin, the enigmatic organizer at GABAergic synapses. Front. Cell. Neurosci. 6:23. 10.3389/fncel.2012.00023 22615685 PMC3351755

[B98] Tretter V. Revilla-Sanchez R. Houston C. Terunuma M. Havekes R. Florian C.. (2009). Deficits in spatial memory correlate with modified γ-aminobutyric acid type A receptor tyrosine phosphorylation in the hippocampus. Proc. Natl. Acad. Sci. U S A 106, 20039–20044. 10.1073/pnas.0908840106 19903874 PMC2785288

[B99] Tyagarajan S. K. Fritschy J. M. (2014). Gephyrin: a master regulator of neuronal function? Nat. Rev. Neurosci. 15, 141–156. 10.1038/nrn3670 24552784

[B100] Tyagarajan S. K. Ghosh H. Harvey K. Fritschy J. M. (2011a). Collybistin splice variants differentially interact with gephyrin and Cdc42 to regulate gephyrin clustering at GABAergic synapses. J. Cell Sci. 124, 2786–2796. 10.1242/jcs.086199 21807943 PMC3148131

[B102] Tyagarajan S. K. Ghosh H. Yevenes G. E. Nikonenko I. Ebeling C. Schwerdel C.. (2011b). Regulation of GABAergic synapse formation and plasticity by GSK3β-dependent phosphorylation of gephyrin. Proc. Natl. Acad. Sci. U S A 108, 379–384. 10.1073/pnas.1011824108 21173228 PMC3017200

[B101] Tyagarajan S. K. Ghosh H. Yevenes G. E. Imanishi S. Y. Zeilhofer H. U. Gerrits B.. (2013). Extracellular signal-regulated kinase and glycogen synthase kinase 3β regulate gephyrin postsynaptic aggregation and GABAergic synaptic function in a calpain-dependent mechanism. J. Biol. Chem. 288, 9634–9647. 10.1074/jbc.m112.442616 23408424 PMC3617267

[B103] Uezu A. Kanak D. J. Bradshaw T. W. Soderblom E. J. Catavero C. M. Burette A. C.. (2016). Identification of an elaborate complex mediating postsynaptic inhibition. Science 353, 1123–1129. 10.1126/science.aag0821 27609886 PMC5432043

[B104] Varoqueaux F. Jamain S. Brose N. (2004). Neuroligin 2 is exclusively localized to inhibitory synapses. Eur. J. Cell Biol. 83, 449–456. 10.1078/0171-9335-00410 15540461

[B105] Vien T. N. Modgil A. Abramian A. M. Jurd R. Walker J. Brandon N. J.. (2015). Compromising the phosphodependent regulation of the GABAAR β3 subunit reproduces the core phenotypes of autism spectrum disorders. Proc. Natl. Acad. Sci. U S A 112, 14805–14810. 10.1073/pnas.1514657112 26627235 PMC4672772

[B106] Villa K. L. Berry K. P. Subramanian J. Cha J. W. Oh W. C. Kwon H. B.. (2016). Inhibitory synapses are repeatedly assembled and removed at persistent sites *in vivo*. Neuron 89, 756–769. 10.1016/j.neuron.2016.01.010 26853302 PMC4760889

[B107] Wang H. Bedford F. K. Brandon N. J. Moss S. J. Olsen R. W. (1999). GABA_A_-receptor-associated protein links GABA_A_ receptors and the cytoskeleton. Nature 397, 69–72. 10.1038/16264 9892355

[B108] Wang Y. C. Dzyubenko E. Sanchez-Mendoza E. H. Sardari M. Silva de Carvalho T. Doeppner T. R.. (2018). Postacute delivery of GABA_A_ α5 antagonist promotes postischemic neurological recovery and peri-infarct brain remodeling. Stroke 49, 2495–2503. 10.1161/strokeaha.118.021378 30355106 PMC6159671

[B109] Wisden W. Seeburg P. H. (1992). GABA_A_ receptor channels: from subunits to functional entities. Curr. Opin. Neurobiol. 2, 263–269. 10.1016/0959-4388(92)90113-y 1379501

[B110] Yamasaki T. Hoyos-Ramirez E. Martenson J. S. Morimoto-Tomita M. Tomita S. (2017). GARLH family proteins stabilize GABA_A_ receptors at synapses. Neuron 93, 1138.e6–1152.e6. 10.1016/j.neuron.2017.02.023 28279354 PMC5347473

[B111] Yogesha S. D. Sharff A. J. Giovannini M. Bricogne G. Izard T. (2011). Unfurling of the band 4.1, ezrin, radixin, moesin (FERM) domain of the merlin tumor suppressor. Protein Sci. 20, 2113–2120. 10.1002/pro.751 22012890 PMC3302654

[B112] Yuan X. Yao J. Norris D. Tran D. D. Bram R. J. Chen G.. (2008). Calcium-modulating cyclophilin ligand regulates membrane trafficking of postsynaptic GABA_A_ receptors. Mol. Cell. Neurosci. 38, 277–289. 10.1016/j.mcn.2008.03.002 18424167 PMC2350232

[B113] Zacchi P. Dreosti E. Visintin M. Moretto-Zita M. Marchionni I. Cannistraci I.. (2008). Gephyrin selective intrabodies as a new strategy for studying inhibitory receptor clustering. J. Mol. Neurosci. 34, 141–148. 10.1007/s12031-007-9018-6 18008186 PMC2758390

[B114] Zhu S. Noviello C. M. Teng J. Walsh R. M. Jr. Kim J. J. Hibbs R. E. (2018). Structure of a human synaptic GABA_A_ receptor. Nature 559, 67–72. 10.1038/s41586-018-0255-3 29950725 PMC6220708

